# C-Reactive protein gene variants are associated with postoperative C-reactive protein levels after coronary artery bypass surgery

**DOI:** 10.1186/1471-2350-10-38

**Published:** 2009-05-08

**Authors:** Tjörvi E Perry, Jochen D Muehlschlegel, Kuang-Yu Liu, Amanda A Fox, Charles D Collard, Simon C Body, Stanton K Shernan, the CABG Genomics Investigators

**Affiliations:** 1 Brigham and Women's Hospital, Harvard Medical School, Boston, MA, USA; 2 Baylor College of Medicine, Division of Cardiovascular Anesthesia, Texas Heart Institute, St Luke's Episcopal Hospital, Houston, TX, USA

## Abstract

**Background:**

Elevated baseline C-reactive protein (CRP) levels are associated with increased risk for developing cardiovascular disease. Several *CRP *gene variants have been associated with altered baseline CRP levels in ambulatory populations. However, the influence of *CRP *gene variants on CRP levels during inflammatory states, such as surgery, is largely unexplored. We describe the association between candidate *CRP *gene variants and postoperative plasma CRP levels in patients undergoing primary, elective coronary artery bypass graft (CABG) surgery with cardiopulmonary bypass (CPB).

**Methods:**

Using a multicenter candidate gene association study design, we examined the association between seventeen candidate *CRP *single nucleotide polymorphisms (SNPs) and inferred haplotypes, and altered postoperative CRP levels in 604 patients undergoing CABG surgery with CPB. Perioperative CRP levels were measured immediately prior to surgery, post-CPB and on postoperative days (POD) 1–4.

**Results:**

CRP levels were significantly elevated at all postoperative time points when compared with preoperative levels (P < 0.0001). After adjusting for clinical covariates, the minor allele of the synonymous coding SNP, rs1800947 was associated with lower peak postoperative CRP levels (*P *= 2.4 × 10^-4^) and lower CRP levels across all postoperative time points (*P *= 4.8 × 10^-5^). rs1800947 remained highly significant after Bonferroni adjustment for multiple comparisons.

**Conclusion:**

We identified a *CRP *gene SNP associated with lower postoperative CRP levels in patients undergoing CABG surgery with CPB. Further investigation is needed to clarify the significance of this association between *CRP *gene variants and the acute-phase rise in postoperative CRP levels with regard to the risk of adverse postoperative outcomes.

## Background

Human CRP is a highly conserved protein belonging to the pentraxin family of acute phase reactants. Normal baseline plasma CRP levels are ≤1 mg/L in healthy individuals[1]. However, as an acute-phase reactant synthesized in the liver, plasma CRP levels can rise 1000-fold in response to tissue injury or inflammation[2,3], thereby triggering activation of the classical complement pathway[4,5], endothelial cell surface adhesion molecule expression[6], leukocyte and macrophage activation, as well as platelet adhesion[1,7]. The pro-inflammatory effects of CRP may explain the increased risk of developing cardiovascular disease (CVD) associated with persistently elevated baseline CRP levels [8-12].

Environmental factors such as age, gender, smoking, lipid levels, hypertension and body mass index (BMI) have been shown to influence baseline plasma CRP levels in the absence of overt inflammatory stimuli[8,13]. Recent family and population-based cohort studies have found moderate heritability of baseline CRP levels [14-16]. There is now convincing evidence to support the association between *CRP *gene variants and baseline plasma CRP levels, and CVD in ambulatory populations [17-19]. Furthermore, two recently published genome wide association studies have identified several genes, including the *CRP *gene, that influence basal CRP levels[20,21]. However, little is known about the influence of *CRP *gene variants on the acute-phase response of plasma CRP levels. We therefore examined the association between candidate *CRP *gene variants and postoperative plasma CRP levels following primary, elective coronary artery bypass graft (CABG) surgery with cardiopulmonary bypass (CPB).

## Methods

### Study design and population

A candidate gene association study was undertaken to examine the association between *CRP *gene variants and postoperative plasma CRP levels following CABG surgery with CPB. One thousand eighty-two patients scheduled for primary, elective CABG surgery at the Brigham and Women's Hospital, Boston, MA and the Texas Heart Institute, St Luke's Episcopal Hospital, Houston, TX between August 2001 and June 2007 were eligible for analysis. Following Institutional Review Boards approval, informed consent was obtained from each patient prior to enrollment. Exclusion criteria were defined prior to statistical analysis in order to exclude patients with ongoing preoperative myocardial injury or other acute inflammatory processes that may have confounded the relationship between CRP variants and postoperative CRP level. Exclusion criteria included age <20 years (n = 0), recent (<2 weeks) myocardial infarction (MI) by patient history (n = 207), preoperative white blood cell count >10.0/mm^3 ^(n = 255), preoperative cardiac troponin I (cTnI) level >0.17 ng/mL (n = 220), and recipients of leukocyte-rich blood products within 30 days prior to surgery (n = 15). We excluded eight patients with a preoperative acute inflammatory process and elevated CRP levels (> 60 mg/L).

### Study protocol

Preoperative demographic data, environmental risk factors and perioperative surgical and anesthetic management were recorded for all patients. Genomic DNA was isolated from whole blood samples using standard techniques[22]. Plasma CRP concentrations were measured with a competitive format immunoassay, using a monoclonal antibody and labeled CRP, configured on Biosite's Triage device platform (Biosite Inc., San Diego, CA), from plasma drawn at six time points: preoperatively, immediately after termination of CPB, and on the mornings of postoperative days (POD) 1–4. CRP concentrations were calculated using a set of spiked plasma samples as the calibration reference. The analytical sensitivity of the CRP assay is 0.33 μg/mL with precision of 17% over the measurable range of the assay (0.33 – 300 μg/mL).

### Candidate SNP identification and genotyping

In an effort to characterize the *CRP *gene and the surrounding region, we genotyped CRP single nucleotide polymorphisms (SNPs) previously described in the literature[23]. In addition, we genotyped tagging SNPs for the region encompassing the CRP gene including 3000 bp upstream of the transcription start site and 1000 bp downstream of the 3' untranslated region (UTR). Tagging SNPs with minor allele frequencies (MAF) >1% were identified using *Tagger *based on a pairwise r^2 ^tagging threshold of 0.8 (rs3316653, rs3316654, rs2794517, rs3122012, rs3093058, rs2808630, rs3093077, rs2794520, rs876538, rs876537, rs1572970)[24]. Genotyping was carried out using the iPLEX genotyping platform (Sequenom, San Diego, CA). SNPs that were not in Hardy-Weinberg equilibrium (P = 0.001) or with a MAF <1% were excluded from analysis. Patients with greater than 10% missing genotypes were also excluded. For haplotype association analysis, PHASE version 2.1.1[25,26] was used to infer common *CRP *haplotypes (frequency >5%) for every individual.

### Statistical analysis

A linear regression model of clinical predictors of postoperative plasma CRP levels was constructed. Clinically relevant variables and those with a *P *= 0.20 on univariate analysis were entered into the model using stepwise regression. Variables included in the final model were age, gender, race, institution, preoperative CRP level, body mass index (BMI), history of chronic inflammatory disease of the bowel, joints or skin, current smoker, corticosteroid use in the month prior to surgery, HMG-CoA reductase inhibitor use within 24 hours of surgery and administration of a homologous blood product during the perioperative period.

Subsequently, the association between *CRP *gene variants and rank-ordered peak postoperative CRP levels was tested for dominant, additive and recessive genetic models while adjusting for the variables in the clinical model. To gain additional insight as to how *CRP *gene variants influence the temporal profile of CRP levels on POD 1–4, CRP measurements were first logit-transformed to approximate a normal distribution – logit-transformation is applied over other forms of transformation to map a bounded variable to the entire real line as is the case with our CRP measurements. Subsequently a repeated-measure Tobit regression model[27], adjusted for the above-mentioned clinical predictors, was applied to estimate the censored normal distribution of the transformed CRP levels while accounting for the correlated observations on different postoperative days for all patients. The repeated-measure Tobit regression model included a random effect to account for the correlation between repeated measures. Finally, robust standard errors were computed based on Huber sandwich estimators to reduce the leverage exerted by any remaining outlier observation in the parameter estimation. Taking into account the posterior probabilities of each possible haplotypic pair, inferred *CRP *haplotypes were tested for association with CRP levels using the repeated-measure Tobit regression model with the most frequent haplotype (H1) as the reference haplotype. A bootstrapping procedure was applied to estimate the effect of each minor allele or haplotype on postoperative plasma CRP levels, and to mitigate the potential for overfitting[28,29]. CRP concentrations are presented as medians with inter-quartile ranges. A *P *< 0.05 after Bonferroni correction was considered statistically significant. SAS version 9.1.3 (SAS Institute, Cary, NC) was used to build the clinical model, R Statistical Software was used for repeated-measure Tobit regression model [30], and PLINK was used for the peak CRP gene association analysis [31].

## Results

**Figure 1:**
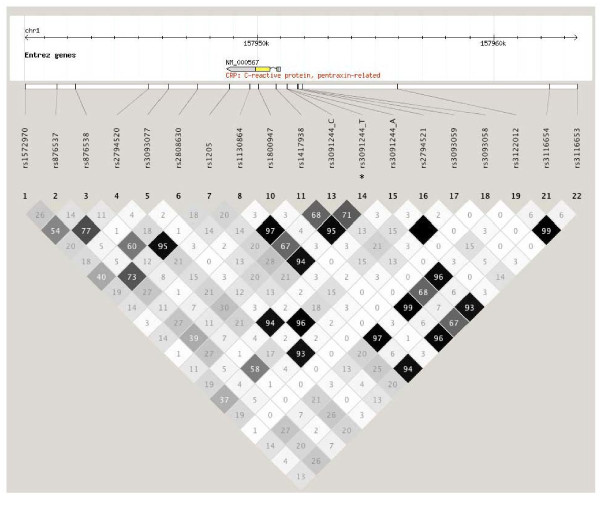
**Linkage disequilibrium (LD) pattern for candidate CRP gene single nucleotide polymorphisms (SNPs)**. NCBI hg version 36. *The triallelic rs3091244 SNP is presented as one base pair apart. Numbers within the pattern represent the r^2 ^values, indicating the level of correlation between single nucleotide polymorphisms. SNPs rs3316653, rs3316654, rs2794517, rs3122012, rs3093058, rs2808630, rs3093077, rs2794520, rs876538, rs876537, and rs1572970 are tagSNPs selected using Haploview's *Tagger *based on a pairwise r^2 ^tagging threshold of 0.8.

After implementing the exclusion criteria, 604 of the original 1082 patients were entered into the analysis. Baseline demographic and clinical characteristics are shown in Table 1. Missing pattern analysis on the excluded patients demonstrated no significant differences, and therefore were considered missing at random. The median preoperative plasma CRP level was 1.2 mg/L IQR [0.6–2.8 mg/L]. Postoperative plasma CRP levels were significantly elevated at all time points compared with the preoperative level (*P *< 0.0001). Median peak postoperative plasma CRP level was 293.3 mg/L IQR [153.6–300.0 mg/L], occurring on POD 2 or 3 in 80.5% of patients. Plasma CRP levels of ≥300 mg/L were measured immediately post-CPB and on PODs 1–4 in 0%, 6.5%, 31.1%, 29.8% and 18.2% of the patients, respectively. Of the nineteen *CRP *SNPs genotyped, two SNPs (rs3093066 and rs3093058) were excluded for MAFs <1%. The remaining seventeen *CRP *SNPs survived predefined genotyping quality control criteria, and were included in subsequent analysis. *CRP *SNPs are characterized in Table 2. The linkage disequilibrium (LD) pattern between genotyped CRP SNPs is shown in Figure 1. All SNPs were in Hardy-Weinberg equilibrium.

**Table 1:** Baseline demographic and clinical characteristics of the study population (n = 604)*.

***Demographic data***	
Age at enrollment (Yrs)	65 ± 10

Gender (Male %)	82

Race (Caucasian vs. Other %)	89

Institution A, (%)	79

***Preoperative Data***	

Body mass index (kg/m^2^)	28.8 ± 4.9

History of diabetes (Types I and II) (%)	28.5

^a^History of peripheral vascular disease (%)	9.7

^b^History of chronic inflammation (%)	16.8

Current smoker (%)	9.3

^c^Preoperative CRP level (mg/L)	1.2 [0.6–2.8]

Preoperative creatinine (mg/dL)	1.09 ± 0.30

***Preoperative Medications***	

^d^Preoperative steroid use (%)	2.0

Duration of statin use prior to CABG (%)	
No statin use	23.1
<1 month	11.1
1–6 months	8.3
≥ 6 months	43.6
Unknown	13.9

Last statin dose administered prior to CABG (%)	
<24 hours	62.1
24–72 hours	5.4
≥ 72 hours	1.3
Unknown	8.1

Preoperative aspirin use (%)	76.3

***Intraoperative Data***	

^e^Blood product transfusion (%)	56.3

Aortic cross clamp time (min)	73 ± 34

Cardiopulmonary bypass time (min)	98 ± 43

Number of coronary grafts (%)	
1	2.8
2	13.8
3	44.9
≥ 4	38.5

*Continuous data are expressed as mean ± SD. ^a^Peripheral vascular disease was defined as vascular disease of the upper or lower extremities, or thoracic or abdominal aorta by patient history. ^b^Chronic inflammation disease of the bowel, joints or skin by patient history. ^c^CRP level expressed in median mg/L with 25^th ^and 75^th ^percentiles.^d^Steroids given by any route within the last month. ^e^Blood product of any kind given intraoperatively through postoperative day 2.

**Table 2:** Candidate CRP gene single nucleotide polymorphisms.

**dbSNP rs#**	**Chromosome Position**^**†**^	**Nucleotide Position**^**††**^	**Role**	**Minor:Major Allele**	**MAF (%)**
rs1572970	chr1:157940209	10691	3' Downstream	G:A	0.287

rs876537	chr1:157941557	9343	3' Downstream	T:C	0.393

rs876538	chr1:157942341	8559	3' Downstream	A:G	0.186

rs2794520	chr1:157945440	5460	3' Downstream	T:C	0.337

rs3093077	chr1:157946260	4640	3' Downstream	G:T	0.071

rs2808630	chr1:157947492	3408	3' Downstream	C:T	0.265

rs1205	chr1:157948857	2043	3'UTR	T:C	0.342

rs1130864	chr1:157949715	1185	3'UTR	T:C	0.299

rs3093066	chr1:157949723	1177	3'UTR	A:C	0.009*

rs1800947	chr1:157950062	838	Coding synonymous	C:G	0.071

rs1417938	chr1:157950810	90	Intron	A:T	0.296

rs3091244	chr1:157951289	-390	Promotor	---	0.624 (C) 0.301 (T) 0.075 (A)

rs2794521	chr1:157951720	-821	Promotor	C:T	0.266

rs3093059	chr1:157951760	-861	Promotor	C:T	0.075

rs3093058	chr1:157951939	-1040	Promotor	T:A	0.006*

rs3122012	chr1:157955947	-5048	Promotor	C:T	0.295

rs2794517	chr1:157959329	-8430	Promotor	A:G	0.224

rs3116654	chr1:157962385	-11486	Promotor	C:T	0.123

rs3116653	chr1:157963534	-12635	Promotor	G:C	0.296

^†^NCBI hg version 36. ^††^Nucleotide position given relative to ATG start codon of the *CRP *gene. The alleles of the triallelic SNP rs3091244 are described in the MAF column. *Excluded from further analyses due to minor allele frequency (MAF) <1%.

Male gender (*P *= 0.002), increased BMI (*P *= 0.046) and perioperative administration of leukocyte-rich blood products (*P *= 0.02) were independently associated with increased peak postoperative CRP levels based on the clinical regression model. There was no difference in minor allele frequencies or CRP levels between Caucasians and non-Caucasians. After adjusting for clinical covariates, including race, the T allele of rs3091244 was independently associated with higher peak postoperative CRP levels (*P *= 2.1 × 10^-3^), while the minor allele (C) of rs1800947 was independently associated with lower peak postoperative CRP levels (*P *= 2.4 × 10^-4^) (Table 3). After adjusting for clinical covariates and repeated CRP measurements using Tobit regression analysis, rs3091244 T remained independently associated with higher postoperative CRP levels (*P *= 2.0 × 10^-3^) and rs1800947 C was independently associated with lower postoperative CRP levels (*P *= 4.8 × 10^-5^) for dominant genetic models (Table 3). Similar model fitting results were found for these SNPs with both dominant and additive genetic models (results not shown). rs1800947 C remained independently associated with lower postoperative CRP levels after adjusting for multiple comparisons. Although rs3091244 T was below the unadjusted level of significance, it achieved only borderline significance after adjusting for multiple comparisons.

**Table 3:** CRP gene single nucleotide polymorphisms association results for postoperative plasma CRP levels following coronary artery bypass graft surgery.

**dbSNP rs#**	**Minor Allele**	**β-Coefficient**^**‡**^	**Peak Postoperative** **CRP *P*-value**^**†**^	**Repeated-Measures** **Postoperative CRP** ***P*-value**^**††**^
rs1572970	G	-0.124	0.709	0.180

rs876537	T	-0.107	0.041	0.272

rs876538	A	-0.143	0.606	0.155

rs2794520	T	-0.104	0.004	0.058

rs3093077	G	-0.056	0.931	0.653

rs2808630	C	-0.061	0.898	0.501

rs1205	T	-0.180	0.004	0.068

rs1130864	T	0.232	0.011	9.0 × 10^-3^

rs1800947	C	-0.477	2.4 × 10^-4^*	4.8 × 10^-5^*

rs1417938	C	0.242	0.007	0.006

rs3091244	C	-0.218	0.026	0.105

rs3091244	T	0.274	2.1 × 10^-3^	2.0 × 10^-3^

rs3091244	A	-0.075	0.948	0.529

rs2794521	C	-0.059	0.932	0.535

rs3093059	T	-0.067	0.986	0.584

rs3122012	C	0.253	0.009	0.007

rs2794517	A	0.057	0.962	0.555

rs3116654	C	0.014	0.567	0.903

rs3116653	G	0.249	0.008	0.005

Results shown for dominant genetic model. ^‡^Describes associated effect of the minor allele on postoperative CRP levels based on a Tobit regression model. The same directional effect was seen for *peak *postoperative CRP levels. ^†^Using rank-ordered peak postoperative CRP level adjusted for clinical covariates. ^††^Using repeated measures of logit-transformed postoperative CRP level in a Tobit regression model and adjusting for clinical covariates. *Below the Bonferroni adjusted level of significance for multiple comparisons.

Four common *CRP *haplotypes (frequency ≥ 5%) were derived from the two most informative *CRP *candidate SNPs, rs1800947 and rs3091244, (Table 4). The overall joint test of *CRP *haplotypes on postoperative CRP levels was highly significant after adjusting for clinical covariates, repeated CRP measurements, and accounting for the posterior probabilities of each possible haplotypic pair (*P *= 7.44 × 10^-8^). Compared with the GC haplotype (H1, the most common haplotype), the CC haplotype (H4) was independently associated with lower postoperative CRP levels (*P *= 4.64 × 10^-3^) whereas GT haplotype (H2) was associated with higher postoperative CRP levels (*P *= 0.029).

**Table 4:** Common CRP haplotypes and association results for altered postoperative CRP levels adjusted for clinical covariates following coronary artery bypass graft surgery.

**Haplotypes**	**rs1800947**	**rs3091244**	**Haplotype Frequency**	**Coefficient**^**‡**^	***P*-value**^**†**^
H1	G	C	0.56	Reference	

H2	G	T	0.29	0.21*	0.029

H3	G	A	0.07	-0.03	0.771

H4	C	C	0.06	-0.36*	4.64 × 10^-3^

H Other	--	--	0.02	-0.56	0.808

Results are shown for dominant genetic model. Major and minor alleles, and forward/bottom strand orientation according to NCBI hg version 36 for Caucasian populations. *CRP *SNPs in strong linkage disequilibrium are combined; therefore not all genotyped SNPs are represented. ^‡^Describes associated effect of each haplotype on postoperative CRP levels based on a Tobit regression model. ^†^Taking into account posterior probabilities, the overall joint test for the haplotype block adjusted for clinical covariates and repeated measures for postoperative CRP was significant at *P *= 7.44 × 10^-8^. *The CC haplotype was significantly associated with decreased postoperative CRP levels at *P *= 4.64 × 10^-3 ^whereas the GT haplotype was associated with increased postoperative CRP levels at *P *= 0.029.

**Figure 2:**
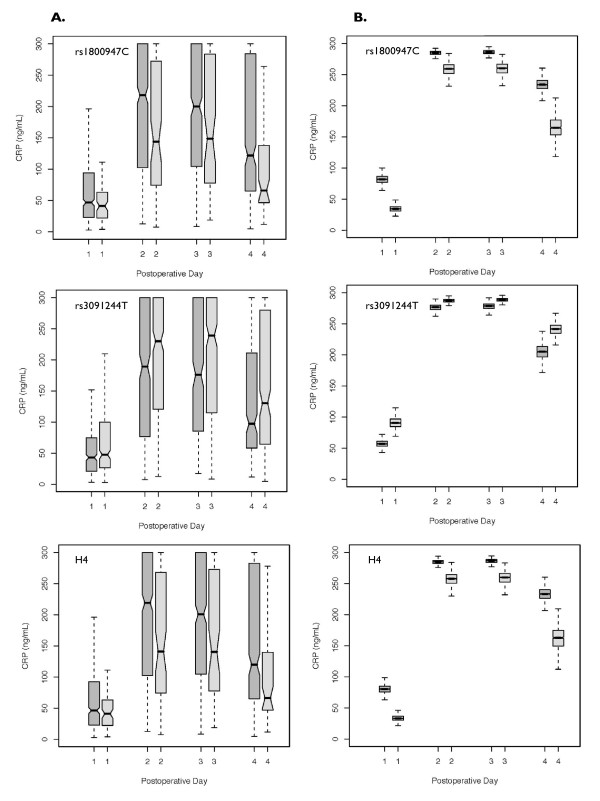
**Observed and estimated genetic associations of rs1800947, rs3091244 T and Haplotype 4 with postoperative plasma CRP levels**. Box plot results are presented for the dominant genetic model. Dark gray denotes null copy, light gray denotes the minor allele of rs180094, rs3091244 T allele and CC haplotype (H4). **Column A**. Observed genetic effect on postoperative CRP levels. rs1800947 minor allele and H4 were associated with lower plasma CRP levels (POD2-4 *P *≤ 0.0095 and *P *≤ 0.0037, respectively), rs3091244 T allele was associated with higher plasma CRP levels (POD2 and 3 *P *≤ 0.0073). **Column B**. Bootstrapping estimates for CRP levels using repeated-measure Tobit regression model adjusted for clinical covariates.

Observed and bootstrapped estimates of the effects of rs1800947, rs3091244 and H4 on plasma CRP levels (POD1-4) are shown in Figure 2. The rs1800947 minor allele was significantly associated with lower plasma CRP levels on POD2-4 (*P *≤ 0.0095), the rs3091244 T allele was significantly associated with higher plasma CRP levels on POD2 and 3 (*P *≤ 0.0073), and H4 was associated with lower postoperative plasma CRP levels on POD2-4 (*P *≤ 0.0037).

## Discussion

We investigated the association between 17 candidate *CRP *SNPs and perioperative plasma CRP levels in patients undergoing primary, elective CABG surgery with CPB. After adjusting for clinical covariates, the minor allele of the synonymous coding SNP, rs1800947 was associated with lower peak postoperative CRP levels, and lower CRP levels across all postoperative time points. Although the rs3091244 T allele did not survive Bonferroni correction for multiple comparisons, this *CRP *SNP was strongly associated with elevated postoperative plasma CRP levels following CABG surgery. Furthermore, these associations were not influenced by race. In the largest cardiac surgical cohort study to date to look at CRP polymorphisms, we provide novel evidence that two *CRP *gene SNPs independently predict postoperative plasma CRP levels following CABG surgery.

The minor allele for the rs1800947 polymorphism has consistently been associated with lower baseline plasma CRP levels[16,19,32-36] and decreased prevalence of CVD-related mortality in ambulatory populations[18,37]. However, the association between *CRP *gene variants and postoperative plasma CRP levels is largely unexplored. Brull *et al*. sought to test the hypothesis that common *CRP *gene variants influence both baseline and postoperative plasma CRP levels in patients undergoing CABG surgery[38]. Possibly limited by low statistical power, these authors failed to demonstrate an association between rs1800947 and increased postoperative CRP levels.

Located on exon 2 of the CRP gene, rs1800947 is a synonymous coding polymorphism. Until recently, it has been thought that synonymous coding polymorphisms, such as rs1800947 (CTG [Leu]→CTC [Leu]), do not affect gene function or phenotype. However, recent evidence suggests that allele-specific differences in codons can result in altered post-translational protein folding and function[39,40]. Nonetheless, without supporting mRNA expression analyses to further elucidate the functional effects of the rs1800947 SNP on plasma CRP levels, we can only speculate as to the functionality of rs1800947. Alternatively, rs1800947 may be in linkage disequilibrium (LD) with a true functional polymorphism; the closest polymorphisms in LD with rs1800947 are rs11265263 (r^2 ^= 0.88) and rs11588887 (r^2 ^= 0.58), 27 kbp and 33.7 kbp in the 5' direction, respectively. Although we were unable to demonstrate significant LD between rs1800947 and other candidate *CRP *SNPs in this study, more extensive genotyping would be necessary to fully exclude LD with surrounding polymorphisms.

rs3091244 is a tri-allelic SNP located in the promotor region of the *CRP *gene that has been associated with elevated baseline CRP levels[19,41-44], and increased risk of coronary artery disease (CAD)[18] in ambulatory patients. However, no study to date has examined the association between rs3091244 and postoperative plasma CRP levels. Located in the second of four identified promotor elements (E-box2) in the *CRP *gene promotor region, Szalai *et al*. recently demonstrated that rs3091244 affects promotor activity *in vitro *by altering transcription factor binding sites[45]. Alternatively, rs3091244 may be a marker allele for a true functional *CRP *gene variant as it is highly correlated with rs3116653, rs3122012, rs1417938 and rs1130864 (r^2 ^≥ 0.93). Of note, rs3091244 is in strong LD with rs1130864 (r^2 ^≥ 0.94), the *CRP *polymorphism described by Brull *et al*. as having a similar effect on postoperative plasma CRP levels in patients undergoing CABG surgery[38]. Further investigation is warranted to determine the importance of rs3091244 in regulating baseline and possibly the postoperative acute-phase rise plasma CRP levels.

The results of our *CRP *haplotype analysis are consistent with our SNP analysis findings. Our result suggests that compared with the GC haplotype (H1), the CC haplotype (H4) was significantly associated with decreased postoperative plasma CRP levels and the GT haplotype (H2) with increased CRP levels. Furthermore, the effect size of the CC haplotype (H4) appeared larger than that of the GT haplotype (H2). This would correspond to two independent modifying effects of the rs1800947C (decrease) and rs3091244T (increase) alleles, consistent with the SNP association findings from Table 3 as H4 was tagged by rs1800947C allele and, H2 tagged by the rs3091244T allele. As such, the results of our haplotype analysis add little additional information to our SNP association findings.

Several limitations in this study warrant consideration. Based on a similar study design, we truncated our plasma CRP measurement at 300 mg/L [38]. In an effort to approximate normal distribution, we logit-transformed the left-skewed postoperative CRP distribution, and applied a Tobit regression to recover the truncated CRP levels. Despite estimated median postoperative CRP levels below 300 mg/L in both groups, the effects of *CRP *gene variants on postoperative plasma CRP levels were large enough to be statistically significant. If we assume that many of the patients with truncated postoperative plasma CRP levels of 300 mg/L in fact had levels greater than 300 mg/L, then our results may in fact underestimate the significance of the association between *CRP *gene variants and postoperative CRP levels after CABG surgery. Genetic association testing of multiple SNPs to CRP levels at many time points in a large sample population also introduces the potential for false positive findings as a result of multiple testing and over-fitting. After applying the Bonferroni correction to address multiple tests of statistical significance, the rs1800947 remained significantly correlated with altered postoperative plasma CRP levels. While rs3091244 did not survive Bonferroni correction, based on prior studies, we believe its biological significance deserves consideration in future investigations. Inherent bias within the sample population can lead to overfitting and false positive results. Bootstrapping is a statistical re-sampling procedure that alleviates the potential for model overfitting by eliminating bias. Our results show that after applying the bootstrapping procedure, the effect of the genetic variants on postoperative plasma CRP levels remained significantly different, therefore making overfitting unlikely. Finally, recently published results from genome-wide association studies suggest that additional proinflammatory genes (*GCKR*, *HNF1A*, *LEPR*, *IL6R*, *APOE*) influence baseline plasma CRP levels in ambulatory populations[20,21]. It is likely therefore, that a number of genes or a combination thereof, may also influence the acute-phase rise in CRP levels.

## Conclusion

Emerging evidence that *CRP *gene variants associated with elevated baseline plasma CRP levels[16,32,36,41] are heritable [14-16]. We submit that the acute-phase rise in postoperative plasma CRP levels after CABG surgery is influenced by *CRP *gene variants. Further investigation is needed to clarify the significance of the association between *CRP *gene variants and the acute-phase rise in postoperative CRP levels with regard to the risk of adverse perioperative outcomes.

## Competing interests

TEP, JDM, KYL, AAF have no competing interests to disclose. CDC, SCB, SKS have received grant support from Biosite, Inc.

## Authors' contributions

TEP, JDM carried out the non-genetic statistical analysis and data interpretation, and drafting of the manuscript. KYL carried out the genetic statistical analysis and interpretation. AAF, CDC made significant contributions to study design and data acquisition. SCB, SKS conceived the study design and analysis approach. The manuscript has been critically revised and approved by all authors.

## Pre-publication history

The pre-publication history for this paper can be accessed here:


